# Hoffmann Reflex Measured From Lateral Gastrocnemius Is More Reliable Than From Soleus Among Elderly With Peripheral Neuropathy

**DOI:** 10.3389/fnagi.2022.800698

**Published:** 2022-03-11

**Authors:** Qipeng Song, Mengzi Sun, Kelsey Lewis, Jung Hun Choi, Brad Manor, Li Li

**Affiliations:** ^1^Biomechanics Laboratory, College of Sports and Health, Shandong Sport University, Jinan, China; ^2^Department of Health Sciences and Kinesiology, Georgia Southern University, Statesboro, GA, United States; ^3^Biomechanics Laboratory, Beijing Sport University, Beijing, China; ^4^Department of Mechanical Engineering, Georgia Southern University, Statesboro, GA, United States; ^5^Hinda and Arthur Marcus Institute for Aging Research, Hebrew SeniorLife, Boston, MA, United States; ^6^Harvard Medical School, Boston, MA, United States; ^7^Division of Gerontology, Department of Medicine, Beth Israel Deaconess Medical Center, Boston, MA, United States

**Keywords:** H-reflex, peripheral nerve, postural control, postural balance, central nervous modulation, peripheral nervous system diseases

## Abstract

**Introduction:**

Peripheral neuropathy (PN) affects up to 20% of the population over the age of 60. Hoffmann reflex (H-reflex) may assess PN adaptation by measuring the function of the peripheral neural system and central nervous system (CNS) modulation. This project aimed to find a reliable muscle among triceps surae muscles during standing and walking among the PN population.

**Materials and Methods:**

Sixteen older adults (> 65 years of age) diagnosed with PN were recruited in this study. The H-reflex test was conducted on the muscle belly of the soleus (SOL), the medial (MG), and lateral gastrocnemius (LG) during standing and walking (heel contact, midstance, and toe-off phases). All measurements were collected on two occasions, separated by at least 7 days. Intraclass correlation coefficients (ICCs) and their confidence intervals (CIs) were used to examine the consistency of the H-reflex outcome variables in the repeated tests for all three tested muscles.

**Results:**

The ICCs of H-index during standing and the three walking phases were poor to moderate in SOL (0.486∼0.737) and MG (0.221∼0.768), and moderate to high in LG (0.713∼0.871). The ICCs of H/M ratio were poor to moderate in SOL (0.263∼0.702) and MG (0.220∼0.733), and high in LG (0.856∼0.958).

**Conclusion:**

The H-reflex of LG was more reliable than SOL and MG during standing and walking among older adults with peripheral neuropathy. It is crucial for future studies in this population to study H-reflex of LG, not SOL and MG, for more reliable results.

## Introduction

Peripheral neuropathy (PN) is estimated to affect 20% of the population over 60 (Suzuki, 2013). As a neurodegenerative disease, PN damages the nerve endings, axons, and myelin of peripheral nerves in a distal to proximal manner (Azhary et al., 2010). Patients often exhibit neural impairments and associated abnormal sensations, including tingling, pricking, burning, and numbness in the lower extremities (Richardson, 2002). Together, these impairments and symptoms decrease postural control (Dixit, 2015), increase the likelihood of falls in older adults (Richardson et al., 1992), along with increased mortality (Kan et al., 2009). Although PN is viewed as a peripheral nerve pathology, the central nervous system (CNS) modulation occurs with the progress of the disease. The CNS alters the latency and amplitude of the monosynaptic stretch reflex by modifying the sensitivity and threshold of excitability of the spinal interneurons (Li et al., 2019).

One of the most common assessments of PN is sensory nerve conduction velocity (NCV), which tests the velocity and amplitude of action potentials in peripheral sensory fibers, most typically the sural nerve (Richardson, 2002). Although decreased sural NCV is the leading assessment of sensory nerve impairment (Richardson, 2002), PN often results in significant sural nerve fiber density degeneration without a decrease in sural NCV (Periquet et al., 1999). Sensory NCV may thus not adequately assess the degeneration of peripheral sensory fibers (Li and Manor, 2010; Grewal et al., 2015), the level of CNS modulation that occurs in the presence of peripheral nerve deterioration, nor the impact of this disease on the function within this vulnerable population.

Hoffmann reflex (H-reflex) has traditionally been used as a window to look into how the CNS modulates the peripheral nervous system. The H-index represents the latency of the monosynaptic peripheral sensory fibers reflexive loop (Knikou, 2008). It is an essential diagnostic tool in neurological impairments because it provides an estimated conduction velocity of an entire monosynaptic reflex arc (Zhang et al., 2015). The H/M ratio between the amplitudes of the maximum reflex response and the maximum direct motor wave is commonly used to estimate the reflex excitability level of the motor neuron pool (Mynark, 2005; Zhang et al., 2015). It helps elucidate the effect of somatosensory reweighting on postural control in many participants with PN vs. healthy individuals with impaired sensation (Li et al., 2019).

Differences between postures may be susceptible to PN-related changes during weight-bearing activities. The amplitude modulation of the H-reflex may vary between different forms or phases of locomotion among older adults with PN. The H-reflex was up to 3.5 times during standing than during walking, and during walking (Capaday and Stein, 1986; Simonsen et al., 2013) or running (Capaday and Stein, 1987; Simonsen et al., 2013), the H-reflex increased progressively during the stance phase, and decreased rapidly at the end of the stance phase and was absent during the swing phase. Soleus (SOL) H-reflex has been proven reliable during standing or walking among healthy individuals (Simonsen and Dyhre-Poulsen, 2011). However, the changed peripheral nerve properties caused by PN may influence H-reflex outcomes (Mynark, 2005; Grewal et al., 2015; Zhang et al., 2015). PN has a progression route from the distal to the proximal direction (Li et al., 2019), so gastrocnemius may have better reliability since it is physically more proximal to the SOL.

Somatosensory reweighting occurs with diseases such as PN, and H-reflex can help to elucidate the impact of somatosensory reweighting on postural control among patients with PN. An intact somatosensory system provides the most accurate information to assist postural control, but it has been established that alternative sources of sensory information can be used to compensate for those who have been impaired (Merabet and Pascual-Leone, 2010). Among patients with PN, proprioception can compensate for their impaired cutaneous sensation (Dixit et al., 2016). Proprioception could be improved by exercise (Zhang et al., 2021) or stretching (Song et al., 2020), identifying the somatosensory reweighting among patients with PN during different postures can help find appropriate rehabilitation methods for this population. To our best knowledge, no studies measured test-retest reliability during standing or walking among older adults with PN. The purpose of this study was to examine the test-retest reliability of H-reflex testing in SOL, MG, and LG during both standing and walking conditions among older adults with PN.

## Materials and Methods

### Participants

Sixteen volunteers (6 women, 10 men, age: 72.3 ± 5.2 years, height: 170.4 ± 10.4 cm, body mass: 91.0 ± 22.6 kg) participated in this project. Inclusion criteria were: (1) age 65 years and older; (2) physician-diagnosed peripheral neuropathy; and (3) impaired foot sole cutaneous sensation as defined by the inability to detect the 5.07-gauge monofilament at three or more of 10 total tested sites (five on each foot sole). Individuals were excluded with one or more of the following: (1) Self-reported history or evidence of central nervous system dysfunction; (2) self-reported trauma or disease that may significantly affect gait or postural control; (3) evidence of foot sole ulcer(s); (4) with a cardiac pacemaker; (5) global cognitive impairment defined by a Mini-Mental State Exam (MMSE) score < 24; and (6) contraindications to physical activity determined by the Physical Activity Readiness Questionnaire Plus (PAR-Q+), i.e., (a) heart condition; (b) high blood pressure; (c) spinal cord disease; (d) lose balance because of dizziness or lost consciousness within the past 12 months; (e) bone, joint, or soft tissue problem that could be made worse by becoming more physically active; (f) only do medically supervised physical activity. An apriori power analysis (G*Power Version 3.1) indicated that a minimum of 11 participants is needed to obtain the alpha level of 0.05 and the beta level of 0.80 based on our previous report (Zhang et al., 2015). All individuals gave written informed consent before participating in the study. Human participation was approved (Nov. 11, 2019) by the University Institutional Review Board (Chair Person: Dr. Andrew Hensen) of Georgia Southern University (H20076) and was in accordance with the Declaration of Helsinki.

### Protocol

All testing procedures were completed during two visits to the lab, with at least 1 week in between. On the first visit, after providing informed consent, individuals completed a medical history, MMSE, the PAR-Q+, and a test of cutaneous sensation to determine eligibility. Height and weight were then recorded. H-reflex testing was completed during standing and then walking conditions, as described below. During the second visit, H-reflex testing was repeated using identical procedures. The skin temperature at the right gastrocnemius was measured each time before the initiation of the H-reflex tests (IRT0421, Infrared Thermometer, Kintrex, Tx, United States).

### Cutaneous Sensation Test

A foot sole cutaneous sensation test was performed with participants in a supine position on an exam table, with a 5.07-gauge Semmes–Weinstein monofilament (North Coast Medical, Inc., Morgan Hill, CA, United States). The testing sites included the heel, midsole, bases of first/fifth metatarsals, and hallux. A score of “1” was given when a “yes” response accompanied the detected pressure, “0” with a “no” response. Each site was tested three times. Then, the score from each site was added. The site was reassigned to “1” if the total score was two or greater; otherwise, the site was reassigned to “0.”

### H-Reflex Test

**FIGURE 1:**
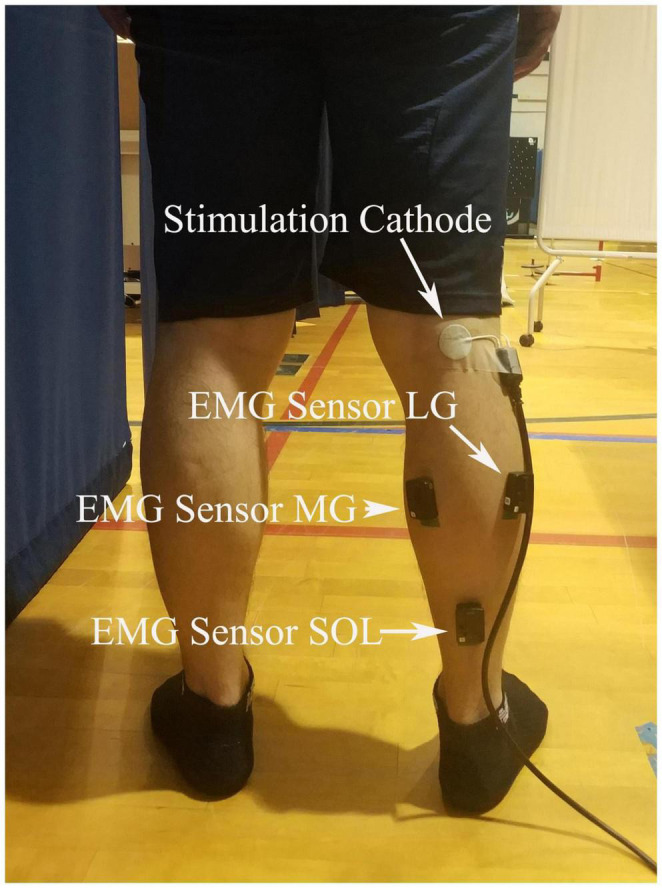
EMG and stimulation locations. Surface electromyography (EMG) electrodes were placed on the muscle belly of soleus (SOL), the medial (MG), and lateral gastrocnemius (LG), and a disposable electrodes cathode was placed on the skin at popliteal fossa.

Surface electromyography (EMG) electrodes (Trigno Wireless EMG System with Avanti Sensors; Delsys Inc., MA, United States) were placed on the muscle belly of SOL, MG, and LG along with the orientation of the muscle fibers (Figure 1). Before the EMG electrodes placement, the skin was shaved and cleaned with alcohol pads. EMG data were collected using EMGWorks (Delsys Inc., MA, United States) at a sampling rate of 2,000 Hz. The optimal site of tibial nerve stimulation in the popliteal fossa (located at the back of the knee) was determined using a hand-held electrode that comes with the stimulator (Digitimer model DS7A, Digitimer Ltd., Welwyn Garden City, England, United Kingdom) at low stimulation intensities. A disposable electrodes (2 cm in diameter) cathode (negative electrode) was placed on the skin at the determined site (Figure 1), and the 5 cm × 8 cm anode (positive electrode) was placed over the patella (kneecap) of the same limb.

**FIGURE 2:**
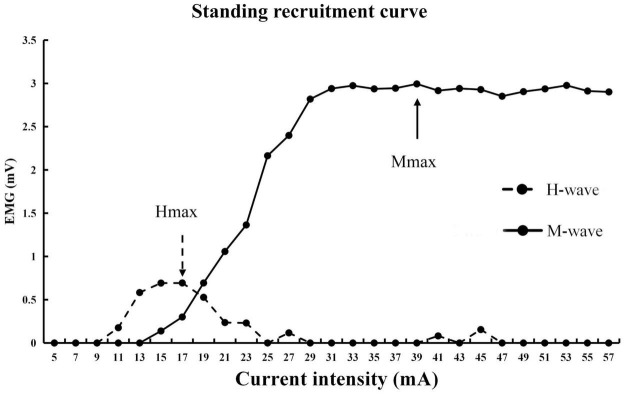
Exemplar H-reflex recruitment curse during standing.

Two testing conditions were used for the H-reflex test, standing and walking. For the standing condition, the standard stimulation protocol was used to estimate H-index and H/M ratio (Chen and Zhou, 2011). During this test, participants were instructed to stand with their feet shoulder-width apart, relax their arms by their sides, and have their vision fixated on a point in their field of view. A 500 μs square-pulse single stimulus elicited H-reflex. Stimulation started at 5 mA and increased with 2 mA increments until the maximum M-wave was reached. Approximately 30 stimuli were executed, with at least 10 s break in between each stimulation. The participants were instructed to limit talking and movement and relax as much as possible during the process. Noise-canceling headphones were used during the test (see Figure 2 for the exemplar recruitment curve). After this test, the participants rested for 10 min or longer upon their request.

**FIGURE 3:**
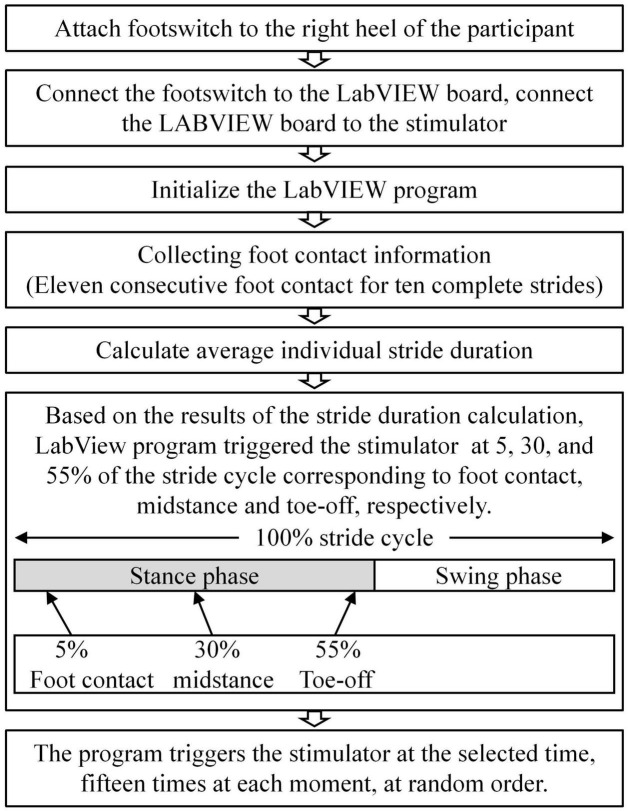
Flowchart of the customized stimulation control during walking using LabVIEW and footswitch. The connection from the footswitch to the LabVIEW board, and from the LabVIEW board to the stimulator were tethered. Heel strike information was detected though footswitch and collected through a customized LabVIEW program at 1,000 Hz. Stride length was calculated from 10 (11) consecutive strides (foot strikes).

Before the walking test, participants completed a 5-min familiarization of treadmill walking at their preferred pace. H-reflex was collected during the three phases in the walking condition, at 5, 20, and 55% of the gait cycle, to represent the heel contact, midstance, and toe-off phases (Querry et al., 2008; Knikou et al., 2011), respectively. A consistent stimuli intensity was applied during all three walking phases, equivalent to the stimulus intensity that elicited 15% of each individual’s maximum M-wave during stance. The stimulation intensity was chosen since it has been demonstrated that M-waves below 15% stimulation level present problems due to the underlying EMG during voluntary muscle activity in the stance phase, and about 25% result in lower H-wave (Simonsen and Dyhre-Poulsen, 1999). The gait cycle was confirmed by a custom-made footswitch system and defined by two consecutive right heel contacts with the ground. A pressure sensor was taped under the right heel of the shoe to detect and record heel contacts during walking. The footswitch system was connected to the stimulator through a LabVIEW board. Heel contact information was detected through footswitch and collected through a customized LabVIEW program at 1,000 Hz. The gait cycle duration was calculated using the average duration of 10 strides before the start of the stimulation. The participants walked on the treadmill for less than 5,min during testing. At least fifteen stimulations were administered in each phase. The H-reflex was estimated using the average of ten recordings for further analysis (see Figure 3 for details).

H-reflex tests were performed during each visit for each participant in a standing-walking fixed order. All measurements were collected on two separate occasions with at least 7 days in between. In this study, a treadmill was used instead of ground walking to improve the safety of participants by using a safety belt fixed above the treadmill and to improve the timing accuracy of the stimulations by using a more consistent walking speed on the treadmill.

### Variables

**FIGURE 4:**
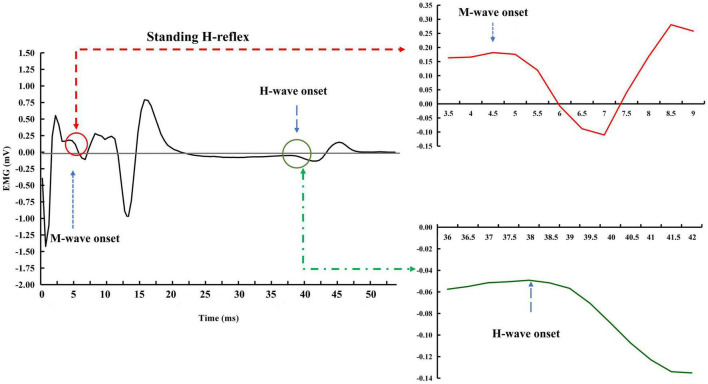
Exemplar H-reflex recordings with standing condition. The onsets of the M- and H-waves were identified based on a single recording. There was a short period of stabled signal in after the stimulation artifact and before the onset of the M-wave. A much more extended period of the stable signal was observed between the end of the M-wave and the beginning of the H-wave.

**FIGURE 5:**
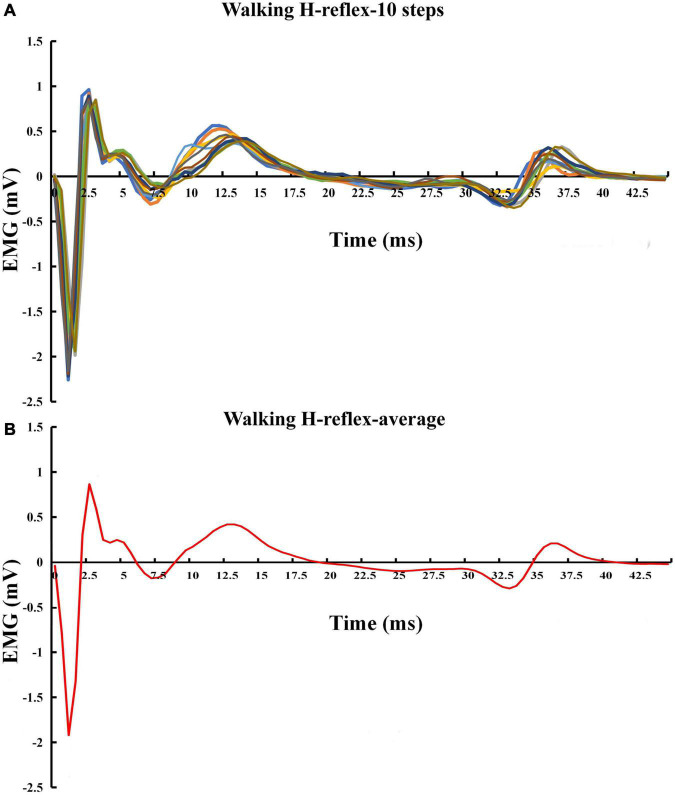
Exemplar H-reflex recordings with walking condition. **(A)** Walking H-reflex in 10 steps. **(B)** Average of the 10 steps.


Raw EMG data were used to estimate H-reflex parameters without filtering and any other types of conditioning. The measurements of interest for this study were the H-index and H/M ratio. H/M ratio during standing was defined as the ratio of magnitudes of maximum peak-to-peak H- and M-waves, i.e., the H/M ratio was as Hmax/Mmax (i.e., the period between the onsets of the H- and M-waves, see Figure 4). H/M ratio during walking was defined as the mean magnitudes of peak-to-peak H-wave across 10 strides over Mmax during standing (Figure 5). H-index was defined as the relative latency between H- and M-waves and was calculated as follows (Li and Manor, 2010):

$$\text{H-index}={\left[\frac{\text{Height}\,\left(\text{cm}\right)}{\Delta \,t_{H}-\Delta \,t_{M}}\right]}^{2}*2$$

### Data Analysis

Statistical analysis was performed using the statistical package SPSS 22.0 (SPSS Inc., Chicago, Illinois). The test-retest reliability of the H-index and H/M ratio was examined using intraclass correlation coefficients (ICC) with a two-way mixed model. The 95% confidence intervals (CIs) of ICCs were also computed. ICC values < 0.60 were considered poor, those between 0.60 and 0.80 were considered moderate, and those > 0.80 were deemed high (Cicchetti and Sparrow, 1981).

## Results

The H-reflex during both standing and walking was successfully elicited from 12 of 16 participants (5 women, 7 men, age: 71.5 ± 4.8 years, height: 170.0 ± 10.0 cm, body mass: 89.3 ± 21.4 kg). In one of these 12 participants (ID#1), the H-reflex was elicited only during walking. The total foot sole sensitivity test score of these 12 participants ranged from 0 to 7 (two scored 0, one scored 1, 4 or 7, respectively, three scored 5, and four scored 6). Skin temperature at the test site was consistent within participants, between the two testing visits, during the tests of both standing (test: 85.5 ± 2.6, retest: 84.7 ± 2.9°F, ICC = 0.60) and walking (test: 84.7 ± 2.9, retest: 85.8 ± 2.7°F, ICC = 0.75).

Table 1 presents the mean and standard deviation, ICCs and their 95%CIs, of the H-index. The ICCs of H-index during standing and heel contact, midstance, and toe-off phases were poor to moderate in SOL (0.486∼0.737) and MG (0.221∼0.768), and moderate to high in LG (0.713∼0.871).

**TABLE 1:** Test-retest reliability of H-index (cm^2^/s^2^), includes the mean, standard deviation (SD), standard error of measurement (SEM), intraclass correlation coefficients (ICC), and 95% confidence intervals (CI) of the H-index.

		Standing		Heel Contact		Midstance		Toe off	
		Test	Re-test	Test	Re-test	Test	Re-test	Test	Re-test
Soleus	Mean	64.2	60.3	63.4	59.6	56.3	59.5	59.3	61.5
	SD	19.9	8.9	10.3	8.8	6.7	7.7	13.2	11.8
	SEM	5.74	2.57	2.99	2.55	1.94	2.22	3.81	3.41
	ICC	0.507		0.486		**0.737**		**0.725**	
	95%CI	–0.065∼0.828		–0.091∼0.819		**0.243∼0.928**		**0.218∼0.924**	
Medial gastrocnemius	Mean	61.4	60.4	60.1	62.0	61.8	63.2	64.2	67.4
	SD	14.26	15.47	10.00	11.04	10.11	9.13	13.88	9.56
	SEM	4.1	4.47	2.89	3.19	2.92	2.63	4.01	2.76
	ICC	0.221		0.223		**0.768**		**0.621**	
	95%CI	–0.378∼0.690		–0.396∼0.713		**0.334∼0.932**		**0.070∼0.882**	
Lateral gastrocnemius	Mean	63.3	64.9	65.4	62.1	65.4	61.5	63.4	61.6
	SD	10.18	10.50	12.47	10.27	12.47	11.13	10.36	9.92
	SEM	2.94	3.03	3.60	2.97	3.60	3.21	2.99	2.86
	ICC	**0.759**		**0.713**		**0.724**		**0.871**	
	95%CI	**0.325**∼**0.929**		**0.265**∼**0.908**		**0.295**∼**0.912**		**0.612**∼**0.961**	

*Bolded ICC and 95% CI indicate moderate or high reliability.*

Table 2 presents the mean and standard deviation, ICCs and their 95% CIs, of the H/M ratio. The ICCs of H/M ratio during standing and heel contact, midstance, and toe-off phases were poor to moderate in SOL (0.263∼0.702) and MG (0.220∼0.733), and high in LG (0.856∼0.958).

**TABLE 2:** Test-retest reliability of H/M ratio, includes the mean, standard deviation (SD), standard error of measurement (SEM), intraclass correlation coefficients (ICC), and 95% confidence intervals (CI) of the H/M ratio.

		Standing		Heel Contact		Midstance		Toe off	
		Test	Re-est	Test	Re-test	Test	Re-test	Test	Re-test
Soleus	Mean	0.32	0.36	0.23	0.26	0.22	0.32	0.249	0.341
	*SD*	0.18	0.22	0.17	0.14	0.15	0.16	0.18	0.43
	SEM	0.05	0.06	0.05	0.04	0.04	0.05	0.05	0.12
	ICC	**0.702**		**0.700**		0.396		0.263	
	95%CI	**0.243∼0.904**		**0.239∼0.903**		–		–0.402∼0.747	
Medial gastrocnemius	Mean	0.11	0.11	0.09	0.09	0.08	0.10	0.08	0.10
	*SD*	0.05	0.06	0.06	0.05	0.05	0.06	0.04	0.08
	SEM	0.01	0.02	0.02	0.02	0.01	0.02	0.01	0.02
	ICC	0.220		**0.666**		**0.733**		0.582	
	95%CI	–0.379∼0.689		**0.179∼0.891**		**0.304∼0.915**		0.010∼0.867	
Lateral gastrocnemius	Mean	0.18	0.21	0.13	0.15	0.12	0.15	0.11	0.12
	*SD*	0.15	0.15	0.13	0.17	0.13	0.14	0.13	0.14
	SEM	0.04	0.04	0.04	0.05	0.04	0.04	0.04	0.04
	ICC	**0.885**		**0.883**		**0.856**		**0.958**	
	95%CI	**0.631**∼**0.968**		**0.645**∼**0.965**		**0.576**∼**0.956**		**0.860**∼**0.988**	

*Bolded ICC and 95% CI indicate high reliability. M-waves used in calculating both H/M ratio during standing and walking were the maximum M-waves for each collection during standing. The H/M ration calculations used maximum H-wave during standing, but H-wave magnitudes during walking were from the stimulation intensive as 15% of that needed for maximum M-wave during standing.*

## Discussion

The present study indicates that h-reflex measured from LG was more reliable than from SOL and MG. Previous studies have reported high reliability in H-reflex in the upper (Stowe et al., 2008) and lower (Mynark, 2005) limb muscles during lying prone and standing among younger and older populations (Mynark, 2005). This study further indicated that the H-reflex in LG, rather than MG and SOL, can be reliably measured during standing and walking among older adults with peripheral neuropathy, despite chronic PN pathology and related CNS adaptation in this population.

In the present study, 75% (12/16) of participants successfully elicited the H-wave. The age, height, and body mass of the four exceptions were within the range of the others, yet they had worse foot sole cutaneous sensation (i.e., lower total foot sole sensitivity test score; median: 1 of the 4 vs. 5 of the 12). The eliciting rate of H-wave in this study is fully consistent with a previous study among older adults with PN (75%, 12/16) (Zhang et al., 2015). Among healthy older adults, the rate was 79% (11/14) (Scaglioni et al., 2002) to 90% (18/20) (Chen et al., 2015), and among healthy middle-aged (Raffalt et al., 2015) or young (Chen et al., 2015) individuals, the rate was 100%. Antidromic action potential could be one reason that partially explained the unsuccessful trials (Palmieri et al., 2004). Other mechanisms by which H-waves cannot be excited in each individual are unknown. It is inferred from the limited information above that the inability of the four participants in our study to excite H-waves was due to their aging and PN. Loss of myelinated and unmyelinated nerve fibers has been reported in the elderly (Verdu et al., 2000), and PN further damaged the axons and myelin of peripheral nerves (Li et al., 2019). The overlapping damage to the peripheral nervous system among older adults with PN may be associated with their low H-wave excitation rates. The H-reflex intensity was determined using approximately 30 stimulations during one standing testing trial. Then the walking trial data were estimated using ten trials. More testing trials during standing and walking can potentially increase the reliability of our observations. However, our participants were elderly with PN, and more trials could lead to exhaustion and failed data collection sessions.

H/M ratio modulation is highly correlated with postural sway and postural stability among adults (average age = 31) (Tokuno et al., 2008). It decreased with increased body sway during the stance phase of walking (Earles et al., 2000) and was amplified after balance training (Taube et al., 2008). The inhibition mechanisms associated with the H/M ratio are not fully understood yet, but are believed to occur at the presynaptic level (Chen and Zhou, 2011). The effect of presynaptic inhibition decreases the H/M ratio under challenging conditions to prevent the overdrive of motor fibers’ autogenic excitation and alter the saturation of motor fiber excitability for receiving CNS commands (Chalmers and Knutzen, 2002). The ability to maintain posture control is related to the complex interactions between peripheral sensory input and motor process modulated by CNS; the functional adaptation of CNS may explain the postural control improvement after different exercise training (Li and Manor, 2010; Grewal et al., 2013, 2015) to training tasks (Taube et al., 2008). From this perspective, the H-reflex in LG, which assesses the function of sensory and motor fibers, CNS modulation, and the influence of postural control, should be better used as a functional assessment tool for older adults with PN.

The LG H-reflex was much more reliable than that of the MG and SOL, especially during walking. LG, MG, and SOL muscles’ mechanical behaviors are different during walking (Orendurff et al., 2005). Aging differentially affects the triceps surae muscles, with age-related network deficits of 38% for the gastrocnemius and 66% for the SOL in all walking speeds. Ebrahimi et al. (2020) inferred that the musculoskeletal function of the SOL is impaired more rapidly with age than that of the gastrocnemius. Moreover, most of the PN has a progression route from distal to proximal direction (Li et al., 2019), i.e., starting from the nerve ending at the bottom of the big toe and growing upwards. Our participants may have different degrees of PN, where cases of SOL nerve ending impairments exist, but the LG nerve endings for most participants were intact. This and other potential mechanisms should be explored further in the future to understand the differential effects of aging and PN on triceps surae H-reflex.

It is worth noting that the reliability of the H-reflex measured from the triceps surae is improved partially due to the advancement of the technology. Compared to the stimulator used before (e.g., Zhang et al., 2015), the present study used a 0.5 ms square wave from a constant current stimulator. They have measured the H-reflex reliability of the LG muscle in a prone position for the same population. The reliability of the H/M ratio was poor, although the H-index reliability is comparable to the current project. The shorter stimulation time (0.5 vs. 1 ms) potentially reduced the possibility of antidramatic interference with the reflexive responses. The constant current (not controllable in the cited paper) provided less variability in the stimulation signals than the previous technology.

This study has several limitations. Antidramatic action potential could be one of the reasons for failing to observe H-reflex among 4 of the 16 participants during walking. And also, we have limited the numbers of our testing trials (one standing and 10 walking) due to the advanced age of our participants with PN. More trials could potentially increase the reliability of the observations. Furthermore, the footswitch sensor was taped under the right heel of the shoe to detect heel contact during walking. However, the shapes and texture of the participant’s shoes were different, leading to inconsistent heel contact detection. Standard testing shoes for the participants in future projects would help to avoid the potential problem. However, the participants in this project wore the same pair of shoes for the test-retest; the results of the reliability examination should not be affected. Finally, the 5-min treadmill walking allotted for each participant to familiarize them with the treadmill setting may not be sufficiently long for an older adult who has never walked on a treadmill before. The familiarization period should only end after the participants feel comfortable walking on the treadmill. A more extended resting period might also be needed if the familiarization led to potential fatigue.

## Conclusion

The H-reflex in LG could be reliably measured. LG provides the most reliable window than the MG and traditional SOL on central modulation of the peripheral nervous system through H-reflex during standing and walking among older adults with peripheral neuropathy.

## Data Availability Statement

The original contributions presented in the study are included in the article/supplementary material, further inquiries can be directed to the corresponding author/s.

## Ethics Statement

The studies involving human participants were reviewed and approved by Institutional Review Board (Chair Person: Dr. Andrew Hensen) of Georgia Southern University (Approval #H20076). The patients/participants provided their written informed consent to participate in this study.

## Author Contributions

QS, MS, and KL participated in the design of the study, contributed to data collection, data reduction and analysis. JC participated in the design of the study and contributed to data collection. BM participated the design and interpretation of results of the study. LL participated in the design of the study, contributed to data analysis, and interpretation of results. All authors contributed to the manuscript writing, read and approved the final version of the manuscript and agreed with the order of presentation of the authors.

## Conflict of Interest

The authors declare that the research was conducted in the absence of any commercial or financial relationships that could be construed as a potential conflict of interest.

## Publisher’s Note

All claims expressed in this article are solely those of the authors and do not necessarily represent those of their affiliated organizations, or those of the publisher, the editors and the reviewers. Any product that may be evaluated in this article, or claim that may be made by its manufacturer, is not guaranteed or endorsed by the publisher.
